# Gut microbiota: a novel target for exercise-mediated regulation of NLRP3 inflammasome activation

**DOI:** 10.3389/fmicb.2024.1476908

**Published:** 2025-01-06

**Authors:** Jun Chen, Shaohui Jia, Xinxuan Xue, Chenggeng Guo, Kunwei Dong

**Affiliations:** ^1^School of Graduate of Wuhan Sports University, Wuhan, China; ^2^School of Sports Medicine of Wuhan Sports University, Wuhan, China; ^3^School of Sports Training of Wuhan Sports University, Wuhan, China; ^4^School of Art of Wuhan Sports University, Wuhan, China

**Keywords:** NLRP3 inflammasome, exercise, gut microbiota, inflammation, immune regulation

## Abstract

The NOD-like receptor family pyrin domain-containing 3 (NLRP3) is a key pattern recognition receptor in the innate immune system. Its overactivation leads to the production of pro-inflammatory cytokines, such as IL-1β and IL-18, which contribute to the development and progression of various diseases. In recent years, evidence has shown that gut microbiota plays an important role in regulating the activation of NLRP3 inflammasome. Variations in the function and composition of gut microbiota can directly or indirectly influence NLRP3 inflammasome activation by influencing bacterial components and gut microbiota metabolites. Additionally, exercise has been shown to effectively reduce NLRP3 inflammasome overactivation while promoting beneficial changes in gut microbiota. This suggests that gut microbiota may play a key role in mediating the effects of exercise on NLRP3 inflammasome regulation. This review explores the impact of exercise on gut microbiota and NLRP3 inflammasome activation, and examines the mechanisms through which gut microbiota mediates the anti-inflammatory effects of exercise, providing new avenues for research.

## 1 Introduction

The nucleotide-binding oligomerization domain-like receptor protein 3 (NLRP3) inflammasome is a key component of the immune system that triggers inflammation and cell death by detecting harmful signals from pathogens or damaged cells, known as ‘danger signals.’ These signals include molecules released from infected or injured tissues, and they play a crucial role in the body’s defense against infection and injury (Broz and Dixit, 2016). A balanced inflammatory response is essential for maintaining host defense function and internal environment homeostasis. However, excessive inflammation from abnormal NLRP3 inflammasome activation is closely associated with the development of inflammatory diseases such as atherosclerosis (AS), type 2 diabetes mellitus (T2DM), inflammatory bowel disease (IBD), and neurodegenerative diseases (Toldo et al., 2022; Sharma and Kanneganti, 2021; Yao J. et al., 2023). Therefore, understanding the mechanisms underlying NLRP3 inflammasome activation and developing targeted therapeutic strategies based on these mechanisms are of significant importance.

The gut microbiota, a large community of microorganisms in the human intestines, participates in food digestion and absorption, and acts as endogenous signaling molecules that regulate the immune system, with researchers considering it a new complex ‘organ’ of the human body (Lavelle and Sokol, 2020). Under normal circumstances, the gut microbiota and the host coexist without causing harmful immune responses. However, when the gut microbiota is disrupted and gut barrier function is impaired, bacterial components and metabolites circulate throughout the body with the bloodstream, inducing NLRP3 inflammasome activation. This leads to chronic inflammatory responses and cascade effects, thereby contributing to the development and progression of multiple diseases (Xing et al., 2023; Pellegrini et al., 2020; Larabi et al., 2020).

Exercise has emerged as a potent intervention that promotes overall health and mitigates the risk of chronic diseases (Khemka et al., 2023). Increasing evidence suggests that exercise exerts beneficial effects on gut microbiota composition and function, promoting a balanced microbial environment that enhances immune regulation (Wegierska et al., 2022). Additionally, exercise has been demonstrated to inhibit NLRP3 inflammasome overactivation induced by various pathological conditions including metabolic disorders, aging, and hypoxia (Zhang et al., 2021). Nevertheless, whether exercise can inhibit NLRP3 inflammasome activation through the regulation of gut microbiota remains to be elucidated. Therefore, this review outlines the effects of different forms of exercise on the gut microbiota and NLRP3 inflammasome activation. It primarily explores how exercise regulates gut microbiota to inhibit NLRP3 inflammasome activation, hoping to provide new target for relevant research.

## 2 Gut microbiota

The gut microbiota is the collective term for the microbial community residing in the human gut, encompassing bacteria, viruses, fungi, and protozoa. A healthy gut microbiota exhibits stability, abundance, and diversity, primarily comprising *Firmicutes*, *Bacteroidetes*, *Actinobacteria*, *Proteobacteria*, and *Fusobacteria*. Within Firmicutes, genera such as *Lactobacillus*, *Bacillus*, *Clostridium*, and *Bacillus* are prominent, while *Bacteroidetes* is dominated by genera like *Bacteroides* and *Prevotella*. Actinobacteria are less abundant, predominantly represented by the genus *Bifidobacterium*. *Firmicutes* and *Bacteroidetes* collectively constitute about 90% of the total gut microbiota (65 and 25%, respectively). The gut microbiota is considered healthy when the *Firmicutes*/*Bacteroidetes* (F/B) ratio is low (Arifuzzaman et al., 2024).

Humans and intestinal flora co-evolve cooperatively: hosts provide nutrients and a reproductive niche for intestinal flora, which in turn helps regulate host physiological functions (Barreto and Gordo, 2023). The gut microbiota participates in energy metabolism and nutrient absorption, and promotes immune system development in the host by producing antimicrobial peptides and other active substances that defend against pathogens (Zong et al., 2020). The gut microbiota is easily influenced by various factors, including host dietary habits, environmental conditions, and intestinal infections. When the intestinal flora is exposed to adverse factors such as environmental pollution, antibiotic abuse, and gastrointestinal diseases, its diversity and stability are compromised. This results in a reduction of beneficial bacteria and an increase in pathogenic bacteria, triggering inflammatory responses and metabolic disorders, ultimately leading to disease (Pires et al., 2024). Numerous studies have shown that the NLRP3 inflammasome pathway plays a crucial role in the inflammatory response caused by the imbalance of intestinal flora homeostasis. Regulating the intestinal flora may be a new direction for targeted therapy of related diseases (Yao H. et al., 2023; Liu C. et al., 2023; Xia et al., 2022).

## 3 NLRP3 inflammasome

### 3.1 Composition and activation of the NLRP3 inflammasome

The NLRP3 inflammasome is a crucial cellular multiprotein complex of the innate immune system, capable of recognizing Damage-associated molecular patterns (DAMPs) or Pathogen-associated molecular patterns (PAMPs) via NLRs (NOD-like receptors) within the PRR family (Swanson et al., 2019). The NLRP3 inflammasome comprises the receptor NLRP3, the adaptor protein ASC, and the effector pro-caspase-1. NLRP3 consists of three domains: an N-terminal pyrin domain (PYD), a C-terminal leucine-rich repeat (LRR) domain, and a central NACHT domain containing an ATPase motif. ASC contains an N-terminal PYD and a C-terminal caspase recruitment domain (CARD). Pro-caspase-1 consists of an N-terminal CARD, a large catalytic subunit called p20, and a small catalytic subunit called p10 at the C-terminus (Xiao et al., 2023).

Upon signal stimulation, NLRP3 oligomerizes through ATP-dependent self-assembly mediated by the NACHT domain. Oligomerized NLRP3 then recruits ASC via homotypic PYD-PYD interactions, assembling into prion-like ASC filaments at clustered PYDs of NLRP3 oligomers. Multiple ASC filaments assemble into a speck, termed the ASC speck (Yu et al., 2024). The C-terminal CARDs of assembled ASC serve as a platform to recruit effector pro-caspase-1 via CARD-CARD interactions, promoting dimerization of the adjacent p20 and p10 catalytic subunits of pro-caspase-1 and self-cleavage to activate the linker between p20 and p10 (Yao J. et al., 2023). Activated caspase-1 cleaves and activates gasdermin-D (GSDMD), leading to the maturation of interleukin-1β (IL-1β) and interleukin-18 (IL-18) cytokines, thereby initiating inflammatory responses (Fu and Wu, 2023).

### 3.2 NLRP3 inflammasome activation pattern

Current research indicates that the NLRP3 inflammasome can be activated via two distinct pathways: the canonical and non-canonical NLRP3 inflammasome activation pathways. Canonical NLRP3 inflammasome activation involves two steps: priming and activation. Normally, cellular NLRP3 levels are low and insufficient to activate the inflammasome. During the priming step, Toll-like receptors (TLRs) and cytokine receptors, such as those for IL-1 and tumor necrosis factor (TNF), activate nuclear factor κB (NF-κB), which promotes the transcription of NLRP3, pro-caspase-1, and pro-IL-1β (Vande Walle and Lamkanfi, 2024). The activation step involves recognizing NLRP3 inflammasome agonists (e.g., crystals, bacteria, and ATP) and assembling and activating the inflammasome. PAMPs or DAMPs induce cellular dysfunctions such as K^+^ efflux, Ca^2+^ influx, mitochondrial dysfunction, reactive oxygen species (ROS) production, mitochondrial DNA (mtDNA) release, and lysosomal disruption. These dysfunctions lead to NLRP3 inflammasome activation and assembly, triggering inflammatory cascade responses (Christgen et al., 2020).

**Figure 1:**
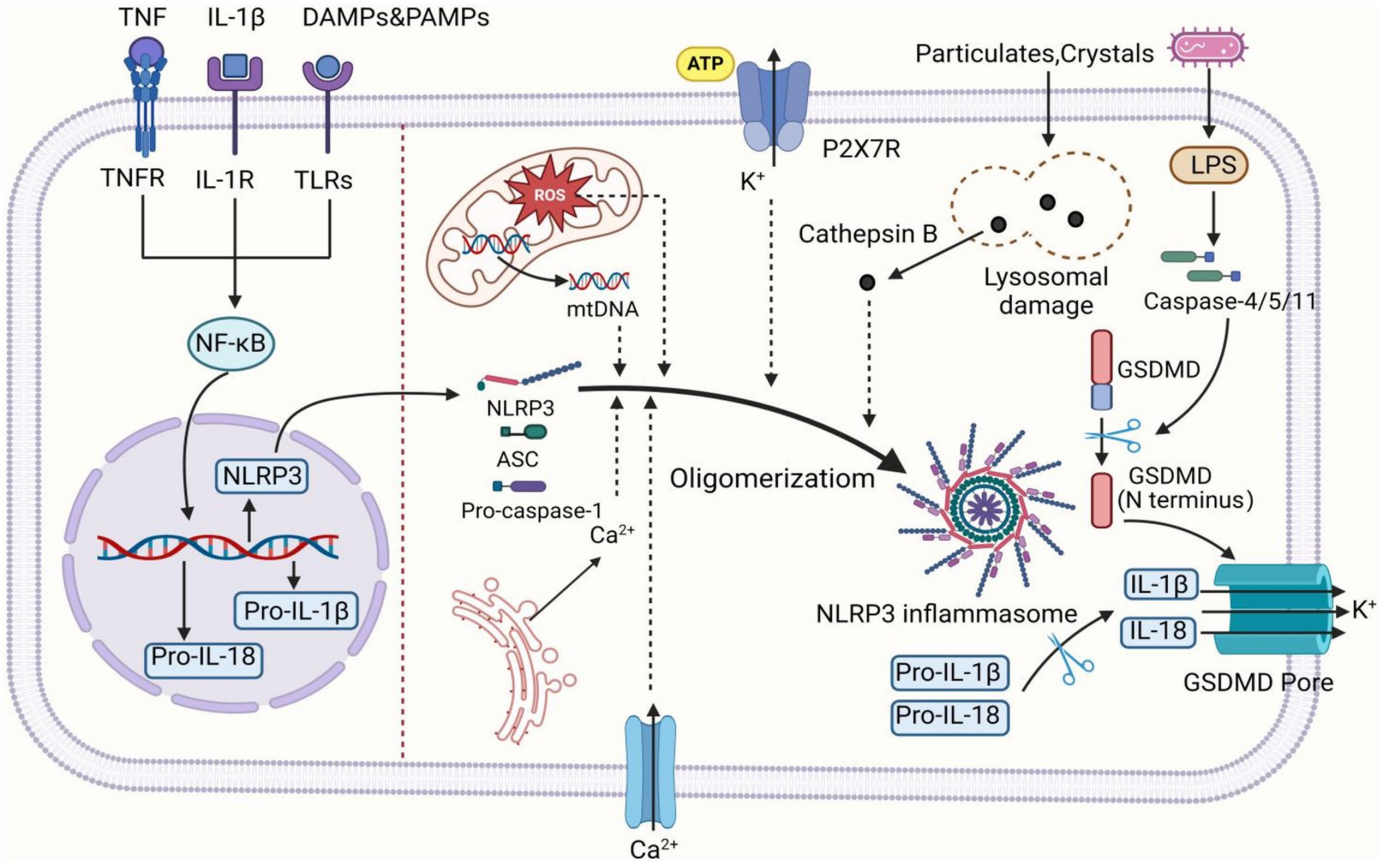
The activation mechanism of the NLRP3 inflammasome. Canonical activation of the NLRP3 inflammasome involves two consecutive signaling steps: priming and activation. During the priming step, various stimuli such as PAMPs, DAMPs, IL-1β, and TNF induce the activation of NF-κB, leading to the upregulation of NLRP3, pro-IL-1β, and pro-IL-18 transcription. In the activation step, NLRP3 can be activated by various upstream signaling events, including K^+^ efflux, Ca^2+^ influx from extracellular and endoplasmic reticulum sources, ROS, mtDNA, as well as lysosomal damage caused by crystals and particles. Upon activation, NLRP3 undergoes oligomerization to serve as a scaffold for recruiting ASC, resulting in the formation of ASC filaments. Subsequently, ASC associates with pro-caspase-1 to assemble into inflammasome. Formation of the inflammasome activates caspase-1, which in turn cleaves pro-IL-1β and pro-IL-18 into mature IL-1β and IL-18. Additionally, caspase-1 can also cleave GSDMD to generate N-terminal and C-terminal domains. The N-terminal domain of GSDMD is released to the cell membrane, forming pores, inducing the intracellular release of IL-1β and IL-18, and triggering pyroptosis. In the non-canonical pathway, upon detecting cytosolic LPS, caspase-4/5 (caspase-11 in mice) is activated and cleaves GSDMD, inducing GSDMD pore insertion into the plasma membrane to promote pore formation. This subsequently triggers K^+^ efflux, thereby activating the NLRP3 inflammasome. The figure was created using BioRender.com.

In non-canonical activation of the NLRP3 inflammasome, Gram-negative bacterial infection releases lipopolysaccharide (LPS) and lipid A into the cytoplasm, causing oligomerization and auto-proteolysis of mouse caspase-11 (human caspase-4/5) precursor. Activated caspase-4/5/11 triggers pyroptosis, inducing K+ efflux and thereby activating the NLRP3 inflammasome (Swanson et al., 2019). Another alternative inflammatory pathway differs from the above two. In response to LPS, human monocytes secrete IL-1β independently of classical inflammasome stimulation. Instead, they propagate inflammatory signals through the TLR4-TRIF-RIPK1-FADD-CASP8 signaling pathway upstream of NLRP3 (Gaidt et al., 2016; Figure 1).

## 4 Exercise and gut microbiota

Exercise can generally be categorized as acute or chronic. Acute exercise is defined as a single, exhaustive physical activity that imposes a significant load on the body, such as marathon running, hiking, and power cycling. This type of exercise induces a stress response in the body, but its effects dissipate quickly. Chronic exercise refers to long-term (4 weeks or more) progressive activities, such as aerobic exercise, resistance training, and high-intensity interval training (HIIT). These exercises induce adaptive changes in body structure and function that persist over time.

Understanding the impact of exercise on gut microbiota is critical, as recent studies suggest that gut microbiota may play a significant role in modulating immune responses (Van Hul and Cani, 2023). Given the association between dysregulated NLRP3 inflammasome activation and various inflammatory diseases, the potential for exercise to modulate this pathway through reshaping gut microbiota represents a promising area of research. In the following sections, we will review the current evidence regarding how exercise influences both gut microbiota and NLRP3 inflammasome activation, focusing on the underlying mechanisms and therapeutic implications.

### 4.1 Acute exercise and gut microbiota

Acute exercise may affect gut microbiota through physiological stress and emergency response mechanisms in the body. It has been suggested that there is a high correlation between physical and emotional stress during acute exercise and changes in the composition of the gastrointestinal microbiota. Intense exercise-induced stress activates the sympathetic-adrenal-medullary axis and the hypothalamic–pituitary–adrenal (HPA) axis, leading to the excessive release of catecholamines (adrenaline and noradrenaline) and other neurotransmitters. These changes induce gastric acid secretion, gastrointestinal motility, and mucin production via the vagus nerve, disrupting gastrointestinal function and potentially disturbing gut microbiota homeostasis (Clark and Mach, 2016). In mouse models, stress from acute exercise reduces the abundance of *Turicibacter* and increases *Ruminococcus gnavus*, *Butyrivibrio*, *Oscillospira*, and *Coprococcus*, exacerbating intestinal inflammation (Clark and Mach, 2016).

Interestingly, gut microbiota changes during acute exercise may help regulate physiological states to adapt to intense exercise stimuli. However, variations in exercise subjects, modalities, and intensities may lead to different changes in gut microbiota. Studies have found that during acute exercise, gut microbiota diversity increases as the body adapts to exercise intensity. The abundance of *Lachnospiraceae*, *Phascolarctobacterium*, and *Akkermansia muciniphila* increased, while *Ruminococcaceae* decreased. These changes may initiate compensatory mechanisms against exercise-induced damage (Bennett et al., 2020).

Keohane et al. documented gut microbiota changes in 4 rowers during different phases (pre, mid, and post) of acute exercise competitions, found that prolonged acute exercise enhances the relative abundance of *Prevotella*, associated with metabolic health and specific microbial metabolic potential, promoting host adaptation to intense exercise stimuli (Keohane et al., 2019). Additionally, Zhao et al. conducted a comprehensive analysis of the fecal metabolites and intestinal flora of amateur half-marathon runners before and after the race. They found that acute exercise accelerates the metabolism of gut bacteria, with the relative abundance of *Actinomycetes* and *Ruminococcus* bicirculans increased, while the abundance of *Ezakiella* and *Romboutsia* decreased (Zhao et al., 2018).

Similarly, Grosicki et al. sequenced the gut microbiota of a world-class ultramarathon runner participating in a 163 km mountain footrace, finding significant increases in gut microbiota *α*-diversity 2 h post-race, accompanied by marked increases in bacterial genera such as *Streptococcus* (+438%) and *Veillonella* (+14,229%) (Grosicki et al., 2019). *Veillonella* can generate energy by metabolizing lactic acid in the gut, while simultaneously reducing lactic acid concentration in the blood and relieving muscle fatigue caused by its accumulation (Ng and Hamilton, 1971). Thus, the increased abundance of *Veillonella* may signify a highly beneficial adaptation to the 163-km race.

From this, it is evident that changes in the composition and metabolic function of the gut microbiota are adaptive responses to acute exercise stress and emergencies. The underlying reason may be that the gut microbiota, as an effective “regulatory organ,” plays a key role in regulating endocrine function and inflammatory response. Based on this, some scholars believe that changes in gut microbiota during acute exercise contribute to regulating motor function. However, relevant research is currently lacking. Therefore, further exploration is needed to investigate the reasons for changes in gut microbiota during acute exercise and the specific mechanisms by which they improve host metabolism. This will contribute to a better understanding of the effects of acute exercise on gut microbiota.

### 4.2 Chronic exercise and gut microbiota

Chronic exercise can prevent and treat several chronic diseases, and the gut microbiota might be involved in many of these beneficial effects. Studies have found that 6 months of combined aerobic and resistance exercise can control diabetes by inhibiting excessive fungal growth in the intestines of T2DM patients, improving intestinal permeability, and alleviating systemic low-grade inflammation (Pasini et al., 2019). Luo et al. shows that chronic moderate swimming can enhance the protein expression of *α*-defensin 5, *β*-defensin 1, regenerating islet derived protein 3β (RegIIIβ), and RegIIIγ, improving intestinal barrier dysfunction induced by chronic stress in mice and reducing microbial translocation (Luo et al., 2014). Allen et al. found that voluntary wheel running and forced treadmill exercise differently affect the gut microbiota of mice at the phylum, genus, operational taxonomic unit (OTU), and α diversity levels. Compared to the forced exercise and sedentary groups, mice engaged in voluntary exercise exhibited a more even distribution of gut microbiota and significantly lower abundance of *Turicibacter* spp., which is closely associated with immune function and intestinal diseases. This suggests that voluntary exercise may be more beneficial for improving gut microbiota health. A 12-week moderate-intensity treadmill aerobic exercise regimen can reverse gut microbiota dysbiosis and increase goblet cell numbers in obese mice. Simultaneously, protein expression levels of ZO-1 and occludin in the colon are elevated, and the adenosine monophosphate-activated protein kinase (AMPK)/caudal-type homeobox 2 (CDX2) signaling pathway is significantly upregulated (Wang et al., 2022). The AMPK/CDX2 signaling pathway promotes the assembly of tight junction (TJ) complexes and plays a key role in improving intestinal barrier function and differentiating of intestinal epithelial cells (IECs). This suggests that aerobic exercise may enhance gut microbiota composition and intestinal barrier function by activating the AMPK/CDX2 signaling pathway.

Additionally, 4 weeks of HIIT can decrease the F/B ratio in insulin-resistant patients, reduce serum levels of TNF-*α*, C-reactive protein (CRP), and lipopolysaccharide-binding protein (LBP), improving systemic insulin resistance (Motiani et al., 2020). Similarly, 4-week resistance exercise can increase the abundance and diversity of gut microbiota in autoimmune encephalomyelitis mice, reduce the F/B ratio and intestinal mucosal permeability, thereby decreasing the inflammatory response of small intestinal lymphoid tissues (Chen et al., 2021). Resistance exercise can reshape the gut microbiota, potentially through its impact on lactate production. Lactate is a byproduct of skeletal muscle glycolysis during exercise. Resistance exercise can lead to lactic acid accumulation, which lowers the pH of the local gut microenvironment. Generally, environmental acidification favors the growth and proliferation of specific microbial communities in the gut, thereby maintaining the stability of the gut microbiota ecosystem (Okada et al., 2013). Wu et al. found that hypoxia-inducible factor 2α (HIF-2α) activates the expression of lactate dehydrogenase, thereby increasing lactate levels in the gut. Lactic acid can induce the expression of the *σ* factor in *Bacteroides vulgaris*, enhance its ability to utilize polysaccharides, and promote the growth and proliferation of *Bacteroides vulgaris* (Wu et al., 2021). Sun et al. reported that supplementation with the lactic acid-producing facultative anaerobic yeast *Saccharomyces cerevisiae* elevates lactate levels in the gut, leading to beneficial changes in microbiota composition and regulating gut homeostasis (Sun et al., 2021). However, some studies have shown that excessive lactate accumulation from gut microbiota may trigger ROS production, adversely affecting intestinal epithelial function (Iatsenko et al., 2018). This suggests that moderate resistance exercise benefits gut microbiota, while excessive exercise may have the opposite effect.

It is important to note that the impact of chronic exercise on gut microbiota can vary depending on the type and intensity of exercise, as well as gender differences. Prolonged high-intensity exercise is known to adversely affect gut homeostasis, resulting in gut microbiota dysbiosis, increased intestinal permeability, compromised intestinal barrier function, and elevated production of inflammatory mediators (Camilleri, 2019). In fact, studies have demonstrated that high-intensity exercise significantly increased gut microbiota associated with elevated gut barrier permeability (e.g., *Firmicutes* and *Actinobacteria*), leading to gut leakage and inflammation. Conversely, moderate-intensity exercise improved gut barrier function by balancing beneficial and harmful bacteria (e.g., *Bacteroides* and *Rikenellaceae*) in a healthy state (Peng et al., 2024). This suggests that exercise intensity can differentially influence gut microbiota composition and function, leading to varied physiological outcomes.

Furthermore, Li et al. conducted a cohort study on aerobic, wrestling, and rowing athletes, finding that rowing athletes had higher intestinal flora Shannon diversity compared to wrestling and aerobic athletes, with no significant differences between the latter two (Li Y. et al., 2023). Interestingly, in the female cohort, *Pseudomonas* and the *Eubacterium coprostanoligenes group* were the most discriminating bacterial groups in aerobic and rowing athlete samples. In the male cohort, *Cryobacillus* and *Bacillus* were the most discriminating genera in aerobic and wrestling athlete samples (Li Y. et al., 2023). These results suggest that both female and male athletes have specialized gut microbiota adapted to their respective sports.

In summary, chronic moderate exercise can positively change the diversity and composition of gut microbiota, thus improve intestinal barrier function, and inhibit inflammatory responses. Conversely, chronic high-intensity exercise may cause gut microbiota dysbiosis, leading to decreased gut barrier function and increased inflammatory responses. Although changes in specific microorganisms vary under different research subjects and exercise types, they may ultimately be to improve body health. Therefore, more studies in this field are needed to clarify the specific regulatory mechanism and relationship between exercise and gut microbiota metabolism. Additionally, when studying the mechanism and relationship between exercise and gut microbiota, besides considering the type, intensity, and duration of exercise, other factors such as diet, sleep pattern, gender, age, initial health status, and antibiotic exposure, should also be considered to avoid these factors impacting the final results.

## 5 Exercise and NLRP3 inflammasome

The impact of exercise on the activation of NLRP3 inflammasome has attracted much attention. Studies have shown that the degree of NLRP3 inflammasome activation varies significantly under different exercise regimens. This variation may be related to factors such as exercise duration (acute vs. chronic), exercise intensity, and individual differences (e.g., training level). The following sections summarize research on the effects of different exercise durations on NLRP3 inflammasome activation (Table 1).

**Table 1:** The effects of exercise on NLRP3 inflammasome activity.

Object	Exercise type/Intensity/Time	Changes of NLRP3 Inflammasome after Exercise	Reference
SD rat	Incremental treadmill running, 8.2–19.3 m/min, 0–10° grade (53–76% VO_2max_), 30–120 min	Myocardial NLRP3 and caspase-1↑	Li et al. (2016)
Healthy young men	Running, Moderate-intensity group: 50–70% HRmax, 70 min; High-intensity group: 70–90% HRmax, 70 min	Moderate-intensity group:PBMCs NLRP3→, serum IL-1β and IL-18→; High-intensity group: PBMCs NLRP3↑, serum IL-1β and IL-18↑	Khakroo Abkenar et al. (2019)
Untrained/trained/triathletes men	Cycle ergometer, 25 W for 2 min (warm-up), 50 W for the second stage, increase by 25 W every 1 min until volitional fatigue	Untrained men:PBMCs NLRP3, caspase-1 and IL-1β↑; trained men:PBMCs NLRP3, caspase-1 and IL-1β→; triathletes men:PBMCs NLRP3, caspase-1 and IL-1β↓	Comassi et al. (2018)
Elderly women	Nordic walking training, 60–70% HRmax, 60 min/d, 3 d/w, 12 weeks	Serum NLRP3 and IL-1β↓	Gomarasca et al. (2022)
Healthy young men	Running, Moderate-intensity: 50–70% HRmax, 70 min, 3 d/w, 3 months; High-intensity: 70–90% HRmax, 70 min, 3 d/w, 3 months	Moderate-intensity group:PBMCs NLRP3↓,serum IL-1β and IL-18↓; High-intensity group:PBMCs NLRP3↑,serum IL-1β and IL-18↑	Khakroo Abkenar et al. (2019)
Obese adults	HIIT, 55–90% HRmax, 35 min/d, 3 d/w, 8 weeks	Serum NLRP3↓	Armannia et al. (2022)
APP/PS1 mice	MICT: treadmill training, 60% Smax, 5 d/w, 12 weeks; HIIT: 85% Smax, 1.5 min; 45% Smax, 2 min, 5 d/w, 12 weeks	Hippocampus NLRP3, ASC, IL-1β and caspase-1↓	Liang et al. (2020)
C57BL/6 J mice	MICT: Treadmill training, 80% SLT, 7 d/w, 4 weeks; HIIT: 60–70% Smax, 5 d/w, 4 weeks	Hippocampus NLRP3↓; HIIT better than MICT	Li et al. (2020)
Healthy older adults	Resistance training, 60–80% 1RM, 3 × 3 sets × 8–12 times, 2 d/w, 8 weeks	PBMCs NLRP3 and caspase-1/pro-caspase-1↓	Mejías-Peña et al. (2017)
Obese adults	Endurance training combined with resistance training, 75% HRmax, 45 min/d, 3 d/w, 4 months	Plasma ASC↓	Barrón-Cabrera et al. (2020)

↑, up-regulation; ↓, down-regulation; →, no significant change; Min, minute; d, day; W, week; VO2max, maximum oxygen uptake; HRmax, maximum heart rate; 1RM, one repetition maximum; Smax, maximum speed; SLT, speed at lactate threshold; HIIT, high-intensity interval training; MICT, moderate-intensity continuous training; NLRP3, NOD-like receptor protein 3; ASC, apoptosis-associated speck-like protein; PBMCs, peripheral blood mononuclear cells; IL-1β, interleukin-1β; IL-18, interleukin-18.

### 5.1 Acute exercise and NLRP3 inflammasome activation

The effect of acute exercise on NLRP3 inflammasome activation may also be related to the body’s stress mechanisms. Studies have found that after acute progressive aerobic exercise, the protein expressions of NLRP3, IL-1β, and mitochondrial autophagy-related genes (Beclin1, LC3, and Bnip3) were significantly upregulated in rat myocardial tissue. The production of mitochondrial ROS and the content of malondialdehyde were significantly increased, along with pronounced inflammatory cell infiltration in myocardial tissue (Li et al., 2016). This suggests that acute progressive exercise induces mitochondrial stress, leading to the activation of the NLRP3 inflammasome, which triggers myocardial inflammatory responses and activates mitochondrial autophagy to reduce myocardial injury in rats. Khakroo et al. found that acute high-intensity aerobic exercise significantly increased NLRP3 mRNA expression in peripheral blood mononuclear cells (PBMCs) and serum IL-1β and IL-18 levels in young healthy men, while acute moderate-intensity aerobic exercise had no significant effect on these indicators (Khakroo Abkenar et al., 2019). This may be due to the lower oxidative stress induced by moderate-intensity exercise, which is insufficient to activate the NLRP3 inflammasome effectively (Powers et al., 2020).

Interestingly, Comassi et al. found that after acute progressive aerobic exercise, the expression of NLRP3, caspase-1, and IL-1β in PBMCs was reduced in endurance athletes. In trained individuals, these indices showed no significant change, while in untrained individuals, their expression increased (Comassi et al., 2018). This indicates that the proinflammatory response induced by acute incremental aerobic exercise may transform into an anti-inflammatory response as training levels increase. This also suggests the existence of exercise preadaptation, where regular long-term exercise allows individuals to better cope with acute stress, such as strenuous exercise, therby inhibit NLRP3 inflammasome overactivation. However, there are few studies on acute exercise, so further research is needed to clarify the mechanisms by which different types and intensities of acute exercise affect NLRP3 inflammasome activation.

### 5.2 Chronic exercise and NLRP3 inflammasome activation

It is a consensus that moderate chronic exercise can effectively inhibit chronic low-grade inflammatory response. Regarding chronic aerobic exercise, moderate-intensity treadmill exercise (60–80% of maximal oxygen uptake or 12–15 m/min) for more than 4 weeks can significantly inhibit NLRP3 inflammasome hyperactivation in rodent heart (Ma et al., 2021; Zhang M. et al., 2023; Kar et al., 2019), blood vessels (Li X. H. et al., 2022; Yang et al., 2023; Lee et al., 2020), cartilage (Liu J. et al., 2023; Yang et al., 2020), liver (Yang et al., 2021; Zhang et al., 2020), kidney (Zhou et al., 2022), hippocampus (Rosa et al., 2021; Liang et al., 2020; Li et al., 2020), lung (Liu et al., 2022), ovary (Weng et al., 2023), and adipose tissue (Mardare et al., 2016; Javaid et al., 2021) caused by metabolic disorders, hypoxia, aging, and other pathological conditions. Additionally, chronic swimming exercise (30 min per day, 5 times per week) (Liu et al., 2015; Bai et al., 2021) and voluntary wheel exercise (averaging 8–12 km per night) (Lee et al., 2018; Zhang et al., 2019) also inhibited the NLRP3 inflammasome overactivation due to depression, high-fat diet, and chronic kidney disease. Similarly, human experiments showed that 12 weeks of moderate intensity nordic walking (60–70% of maximum heart rate) significantly decreased NLRP3 (−53%) and TLR4 (−63%) mRNA expression in serum, as well as IL-6, IL-1β, and TNF-*α* levels in elderly women (Gomarasca et al., 2022). This suggests that moderate-intensity aerobic exercise may reduce TLR4 expression, thereby suppressing NLRP3 inflammasome activation.

The modulatory effect of aerobic exercise on NLRP3 inflammasome activation is related to mitochondrial mechanisms. NLRP3 inflammasome activation is primarily regulated by the suppression of mitochondrial damage and/or the release of mitochondrial signals, leading to an “all-or-nothing” response (Afonina et al., 2017). Aerobic exercise promotes mitochondrial biogenesis, enhances mitophagy, and optimizes mitochondrial dynamics, thereby increasing antioxidant capacity, reducing mitochondrial ROS production, and inhibiting NLRP3 inflammasome activation (Zhang et al., 2021). A study found that 8 weeks of treadmill exercise reduced mitochondrial fusion protein (mitofusin 2, MFN2) expression in the hearts of hypertrophic mice and suppressed the downstream NLRP3/caspase-1/IL-1β signaling pathway, thereby alleviating myocardial hypertrophy and improving cardiac function (Ma et al., 2021). Thus, aerobic exercise may inhibit the NLRP3 inflammasome pathway by enhancing mitochondrial quality control. Additionally, NF-κB, an important regulator of mitochondrial function, plays a crucial role in mediating mitophagy (Shi et al., 2023). Regulating the NF-κB signaling pathway may be key to aerobic exercise’s inhibition of NLRP3 inflammasome activation. For instance, one study demonstrated that 8 weeks of treadmill exercise inhibited acetylation of forkhead box transcription factor O1 (FOXO1) in the brain tissue of diabetic rats, promoted FOXO1 phosphorylation, reduced FOXO1 protein expression, and downregulated NF-κB, TNF-*α*, and NLRP3 inflammasome expression, suggesting that aerobic exercise may suppress inflammation via the FOXO1/NF-κB/NLRP3 inflammasome pathway (Wang et al., 2019). Furthermore, Zhou et al. showed that 8 weeks of aerobic exercise reduced levels of NADPH oxidase 4 (NOX4), ROS, mitochondrial complex I, and protein expression of TNF-α, IL-18, NF-κB p65, IκBα, and the NLRP3 inflammasome in renal tissue of mice, indicating that aerobic exercise may inhibit NLRP3 inflammasome activation by modulating the NOX4/ROS/NF-κB signaling pathway (Zhou et al., 2022).

Notably, the effects of chronic aerobic exercise on NLRP3 inflammasome activation seem to depend on exercise intensity. For example, Khakroo et al. found that 12 weeks of moderate-intensity aerobic exercise significantly reduced NLRP3 mRNA expression in PBMCs and serum IL-1β and IL-18 levels in young men. In contrast, high-intensity aerobic exercise significantly increased these indicators (Khakroo Abkenar et al., 2019). This may be due to high-intensity aerobic exercise increasing *TLR4* gene expression (de Vicente et al., 2020). Therefore, for aerobic exercise, chronic moderate-intensity aerobic exercise might be the optimal regimen to inhibit overactivation of the NLRP3 inflammasome.

In addition to aerobic exercise, HIIT, resistance training, and combined resistance and aerobic exercise also inhibit NLRP3 inflammasome overactivation. Studies have shown that 8 weeks of HIIT can significantly reduce NLRP3 gene expression in the serum of obese adults (Armannia et al., 2022). Liang et al. (2020) and Li et al. (2020) also found that chronic HIIT significantly inhibit NLRP3 inflammasome overactivation in the hippocampus of Alzheimer’s mice and stroke-induced depression mice. However, the former study found that the inhibitory effect of HIIT on NLRP3 inflammasome was not significantly different from that of moderate-intensity continuous training (MICT), while the latter study found that HIIT inhibited NLRP3 inflammasome better than MICT. The inconsistency in results may be due to the large differences in the pathological models of mice, exercise duration, and exercise intensity used in the studies.

Additionally, Mejias-Pena et al. found that compared to a sedentary group, elderly individuals showed significantly lower expression of NLRP3, caspase-1, and apoptosis-related genes (Bcl-2, Bcl-xL) in PBMCs after 8 weeks of resistance training. The protein expression of autophagy-related genes (Beclin-1, Atg12, Atg16, and LAMP-2) was significantly upregulated (Mejías-Peña et al., 2017). This suggests that resistance exercise can inhibit NLRP3 inflammasome activation and reduce cell apoptosis by activating autophagy in PBMCs. Similar to aerobic exercise, resistance exercise can also inhibit NLRP3 inflammasome activation through the regulation of the NF-κB pathway. For example, a study showed that 12 weeks of resistance exercise significantly increased AMPK phosphorylation, silent information regulator 1 (SIRT1), and NLRP3 inflammasome protein expression in the hippocampus of IR mice, while reducing NF-κB expression (Ji et al., 2020). These findings suggest that resistance exercise regulates inflammation through the AMPK/SIRT1/NF-κB/NLRP3 inflammasome signaling pathway.

Moreover, recent studies have demonstrated that 12 weeks of combined with moderate-intensity aerobic and resistance exercise significantly reduces peripheral blood ASC, macrophage inflammatory protein-1 (MCP-1), and MIP-1β mRNA expression in obese adults, and decreases the atherogenic index (Barrón-Cabrera et al., 2020). This suggests that combining aerobic and resistance exercise might inhibit NLRP3 inflammasome assembly and activation by reducing ASC expression.

In conclusion, various types of chronic moderate exercise can inhibit NLRP3 inflammasome overactivation. However, most current studies focus on aerobic exercise, with a lack of comparative research on the effects of different exercise types, intensities, and frequencies on NLRP3 inflammasome activation. Additionally, most human studies are conducted at the systemic level (blood), with fewer at the tissue and organ levels. Therefore, the effects and mechanisms of chronic exercise under different regimens on NLRP3 inflammasome activation in various tissues require further exploration.

## 6 Potential mechanisms of exercise-mediated regulation of gut microbiota on NLRP3 inflammasome activation

Although existing studies have found that exercise can positively regulate gut microbiota and inhibit inflammatory responses, there are currently few reports on the regulation of NLRP3 inflammasome activation by exercise through the gut microbiota. Research has shown that 12 weeks of combined aerobic and resistance exercise can significantly reduce the relative abundance of Proteobacteria and increase the relative abundance of *Blautia*, *Dialister*, and *Roseburia* in obese children, concurrently reduced the protein expression of NLRP3 and caspase-1 in PBMCs (Quiroga et al., 2020). Additionally, Lv et al. found that 4 weeks of voluntary running wheel exercise in mice reduced the expression of NLRP3, caspase-1, IL-18, and IL-1β proteins in brain tissue after ischemic stroke, concurrently reduced *Ruminococcus*, and increased *Lactobacillus* and *Alistipes* abundance, thereby improving cognitive impairment (Lv et al., 2022). These findings suggest that exercise may inhibit NLRP3 inflammasome activation via gut microbiota modulation. However, the specific mechanisms behind this correlation remain unclear. Therefore, this section investigates the potential mechanisms through which exercise modulates NLRP3 inflammasome activation via the gut microbiota.

### 6.1 Exercise reduces bacterial components to inhibit NLRP3 inflammasome activation

LPS is a unique component of the cell walls of Gram-negative bacteria in the gut microbiota. Normally stored in the gut, it is found in low amounts in the bloodstream. When gut microbiota dysbiosis occurs, large amounts of LPS detach from the cell walls of Gram-negative bacteria and activate TLR4 on immune cells in the intestinal wall and its downstream inflammatory signaling pathways, producing large quantities of pro-inflammatory cytokines such as IL-6, TNF-*α*, and IL-1β (Guo et al., 2022). These cytokines can damage intestinal epithelial cells and their tight junctions, leading to increased intestinal permeability, or “leaky gut” (Camilleri, 2019). Additionally, the increased intestinal permeability allows LPS to translocate from the gut into the bloodstream more easily, where it binds to TLR4 on the cell membranes of tissues and organs throughout the body, triggering a systemic inflammatory response (Honda and Inagawa, 2023). Therefore, regulating the gut microbiota to reduce LPS production and its translocation may effectively inhibit NLRP3 inflammasome activation.

Moderate exercise can improve gut microbiota composition and protect the intestinal barrier, reducing LPS production and translocation. Li et al. found that a high-fat diet-induced osteoarthritis mouse model showed reduced gut microbiota diversity, increased endotoxin-producing bacteria, decreased protective gut barrier bacteria, elevated LPS levels in blood and joint fluid, and increased TLR4 expression. However, voluntary wheel running could partially reverse these adverse changes, suggesting that moderate exercise might reduce circulating LPS levels by reshaping the gut microbial ecosystem and improving intestinal barrier function (Li et al., 2021). Wang et al. discovered that 7 weeks of HIIT significantly increased gut microbiota alpha diversity in middle-aged male ICR mice, decreased the relative abundance of *Proteobacteria* (Gram-negative bacteria), and increased TM7 abundance. A Kyoto Encyclopedia of Genes and Genomes (KEGG) analysis showed that HIIT reduced pathways related to LPS biosynthesis and its proteins (Wang et al., 2020). This suggests that HIIT may reduce the production of LPS by decreasing the relative abundance of Gram-negative bacteria and inhibiting the gene pathways related to LPS biosynthesis.

**Figure 2:**
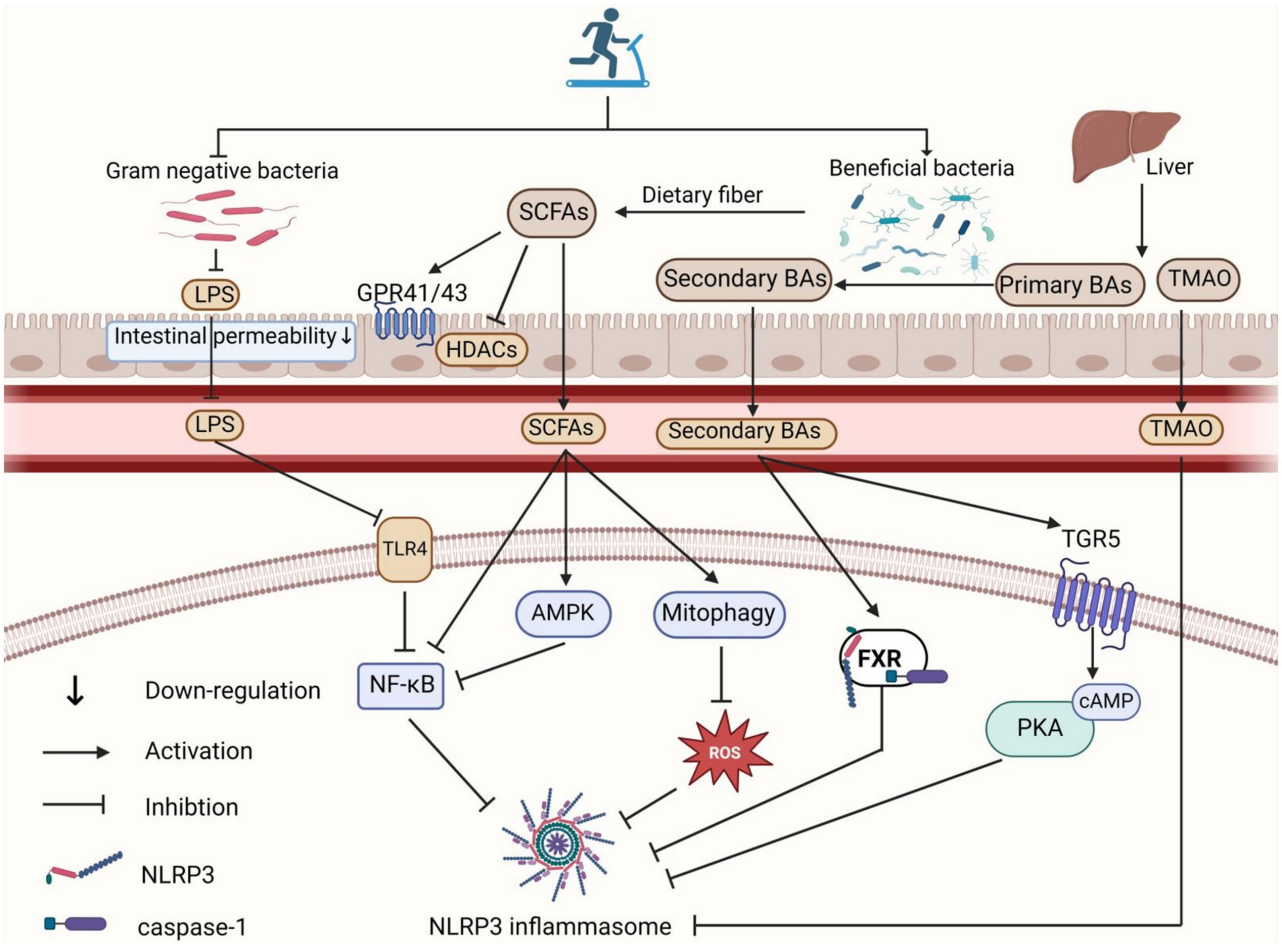
Role of exercise-mediated gut microbiota in inhibiting NLRP3 inflammasome activation. Exercise reduces the proportion of Gram-negative bacteria in the gut microbiota, leading to decreased LPS production and translocation into the circulation, which in turn inhibits the TLR4-NF-κB pathway and suppresses NLRP3 inflammasome activation. SCFAs inhibit NLRP3 inflammasome activation by interacting with GPR41/GPR43 receptors and inhibiting HDAC in intestinal epithelial and inflammatory cells. SCFAs also reduce NLRP3 inflammasome activation through mitochondrial autophagy/ROS and AMPK/NF-κB pathways. Moreover, exercise-induced alterations in the gut microbiota increase secondary BA concentrations, which activate the BA receptors FXR and TGR5 signaling pathways, thereby inhibiting NLRP3 inflammasome activation. Additionally, exercise lowers gut production of TMA, reducing its oxidation to TMAO in the liver, which decreases circulating TMAO levels and inhibits NLRP3 inflammasome activation. The figure was created using BioRender.com.

Given that high-intensity exercise might disrupt gut microbiota, impair intestinal barrier function, and lead to LPS translocation, Peng et al. compared the effects of 12 weeks of HIIT and MICT on gut microbiota and LPS translocation in mice. They found that MICT increased beneficial bacteria, improved intestinal barrier function, and reduced LPS levels in the blood and brain. In contrast, HIIT increased bacteria associated with higher gut permeability, induced “leaky gut,” and resulted in excessive LPS in the blood and brain (Peng et al., 2024). The inconsistent results with HIIT might be due to differences in exercise intensity, frequency, and duration. Therefore, the specific exercise regimens in HIIT warrant further investigation. Overall, moderate exercise might reduce the abundance of LPS-containing Gram-negative bacteria and improve intestinal barrier function by reshaping the gut microbiota, thereby reducing LPS production and translocation, ultimately inhibiting NLRP3 inflammasome activation (Figure 2).

### 6.2 Exercise regulation of gut microbiota metabolites to inhibit NLRP3 inflammasome activation

#### 6.2.1 Exercise regulation of short-chain fatty acids to inhibit NLRP3 inflammasome activation

Short-chain fatty acids (SCFAs) are metabolic byproducts mainly produced by anaerobic bacteria, such as *Lactobacillus* and *Bifidobacterium*, through the fermentation of dietary fibers and glucose. The primary types of SCFAs are acetate, butyrate, and propionate. As important energy sources for the gut microbiota and host intestinal epithelial cells, SCFAs can inhibit the growth of harmful bacteria, reduce inflammation, and help maintain gut microbiota homeostasis and host immunity (Morrison and Preston, 2016). Studies have shown that SCFAs can directly activate G-protein coupled receptors (GPCRs), such as GPR41 (also known as free fatty acid receptor 3, FFAR3) and GPR43 (also known as free fatty acid receptor 2, FFAR2), or enter cells by passive diffusion to inhibit histone deacetylases (HDAC), thereby reducing inflammation and repairing intestinal barrier function (Koh et al., 2016). When SCFA levels are reduced, it leads to decreased metabolite-sensing GPCR binding, impaired intestinal integrity, and the entry of toxic substances like LPS into the bloodstream, thereby activating the NLRP3 inflammasome and causing systemic inflammation (Sun et al., 2017).

The theory that exercise promotes SCFA production to exert anti-inflammatory effects has been validated in numerous studies. Vijay et al. found that a 6 weeks exercise intervention in 78 elderly community members increased the relative abundance of *Bifidobacterium*, *Faecalibacterium*, and *Eubacterium*, raised butyrate production, and significantly decreased levels of inflammatory factors such as TNF-*α* and IL-6 (Vijay et al., 2021). Huang et al. reported that 3 months of endurance exercise effectively increased the abundance of SCFA-producing bacteria, such as *Rikenellaceae* and *Dubosiella*, in the gut of apolipoprotein E (ApoE) knockout mice. This promoted SCFAs production, inhibited the expression of MCP-1, IL-1β, and TNF-α, and ultimately improved atherosclerosis syndrome (Huang et al., 2022). Further research indicated that in high-cholesterol diet mice, voluntary wheel running increased the relative abundance of *Lactobacillus* and *Eubacterium nodatum* in feces, elevated SCFAs content in the cecum, and upregulated GPR109A and GPR41 mRNA expression in the colon, while reducing inflammation-related markers (Li R. et al., 2023). This suggests that exercise may inhibit inflammation by promoting the production of SCFAs by the gut microbiota, which in turn activates GPR41/GPR109A. In an IBD rat model, supplementation with food-derived oryzanol increased SCFAs levels in the gut, which inhibited the TLR4/NF-κB/NLRP3 pathway and reduced intestinal barrier damage and inflammation (Xia et al., 2022). Additionally, in LPS-pretreated bovine macrophages, butyrate treatment inhibited LPS-induced NLRP3 inflammasome activation by suppressing the HDAC/NF-κB signaling pathway (Jiang et al., 2020). Therefore, exercise-induced SCFAs production may inhibit NF-κB signaling and enhance intestinal barrier function through the GPR41/GPR43 and HDAC pathways, thereby inhibiting LPS-induced NLRP3 inflammasome activation.

In addition, exercise-induced SCFAs production may also inhibit NLRP3 inflammasome activation by promoting mitophagy and activating the AMPK pathway. Mitophagy is crucial for mitochondrial quality control, maintaining mitochondrial homeostasis by removing damaged mitochondria and reducing mitochondrial ROS levels (Shen et al., 2023). Studies have shown that supplementing with *Lactobacillus acidophilus* increases SCFAs levels in mice with ulcerative colitis and reduces ROS production by activating mitophagy, which inhibits NLRP3 inflammasome activation and alleviates intestinal inflammation (Li P. et al., 2022). Furthermore, SCFAs can elevate the intracellular AMP/ATP ratio, which leads to AMPK activation (Tao and Wang, 2024). Activated AMPK negatively regulates the IKK/IκB/NF-κB signaling pathway, thereby suppressing inflammation (Sag et al., 2008). In a mouse model of severe acute pancreatitis-associated acute lung injury, oral administration of Qingyi Decoction increased the relative abundance of SCFA-producing bacteria and SCFAs levels in the intestine, serum, and lungs, especially propionate and butyrate, thereby activating the AMPK/NF-κB/NLRP3 signaling pathway and reducing systemic inflammatory responses (Wang et al., 2023). Therefore, it is speculated that exercise-mediated SCFAs production may also inhibit NLRP3 inflammasome activation by activating the mitophagy/ROS pathway and the AMPK/NF-κB pathway.

Notably, different types of SCFAs may have varying effects on NLRP3 inflammasome activation. *In vitro* studies using endothelial cells have shown that butyrate significantly inhibits cholesterol crystal-induced NLRP3 inflammasome activation, whereas acetate not only fails to inhibit this activation but may also activate the NLRP3 inflammasome (Yuan et al., 2018). It is also noteworthy that Torquati et al. (2023) compared the effects of 8 weeks of MICT and HIIT on SCFA-producing gut microbiota in T2DM patients and found that exercise intensity affects SCFA-producing bacteria differently. Under MICT, the relative abundance of butyrate-producing bacteria such as *Bifidobacterium* and *A. municiphila* was higher, while HIIT increased the relative abundance of other butyrate-producing bacteria such as *Erysipelotrichales* and *Oscillospira*. Although exercise intensity did not significantly affect the final SCFA output, it suggests that specific exercise intensities may target particular SCFA-producing bacterial. Given that different SCFAs may play distinct roles in preventing and treating NLRP3 inflammasome-related diseases, and considering the current lack of research comparing the effects of various exercise types, intensities, and frequencies on different types of SCFAs production, future studies should focus on these variables to design more targeted exercise prescriptions for specific diseases (Figure 2).

#### 6.2.2 Exercise regulation of bile acids to inhibit NLRP3 inflammasome activation

Bile acids (BAs) are important components of bile, playing crucial roles in metabolism, immunity, and inflammation regulation (Xiang et al., 2021). Bile acids are classified as primary or secondary. Primary BAs, such as cholic acid (CA) and chenodeoxycholic acid (CDCA), are synthesized in the liver. After food intake, bile acids stored in the gallbladder are released into the intestine. Approximately 95% of BAs are absorbed at the distal ileum and return to the liver via enterohepatic circulation (Gonzalez, 2012). The remaining BAs are substrates for microbial metabolism in the colon, where bacteria with bile salt hydrolase (BSH) and 7α-dehydroxylase convert them into secondary BAs, such as deoxycholic acid (DCA) and lithocholic acid (LCA) (Staley et al., 2017). Dysbiosis of the gut microbiota can lead to disrupted BA metabolism, with increased levels of primary BAs and decreased levels of secondary BAs (Grüner and Mattner, 2021).

Increasing evidence suggests that BAs are crucial in regulating NLRP3 inflammasome activation. Studies have found that during cholestasis, excessive CDCA promotes ROS production and ATP release, inducing intracellular K^+^ efflux and dose-dependently enhancing LPS-induced NLRP3 inflammasome activation in mouse macrophages, leading to IL-1β release (Gong et al., 2016). Additionally, DCA, CDCA, and their taurine-conjugated forms act as DAMPs, inducing prolonged extracellular Ca^2+^ influx and synergizing with ATP to activate the NLRP3 inflammasome in immune cells like peritoneal macrophages (Hao et al., 2017). These studies suggest that BAs can act as endogenous DAMPs or synergize with exogenous PAMPs, thereby inducing the activation of the NLRP3 inflammasome.

Notably, recent studies showed that BAs may have different effects on NLRP3 inflammasome activation under varying inflammatory conditions. Under non-inflammatory conditions, secondary BAs supplementation can activate the NLRP3 inflammasome in THP-1 differentiated macrophages, promoting inflammation. In contrast, in LPS-induced inflammatory macrophages, secondary BAs reduce inflammation by inhibiting NLRP3 inflammasome activation (Liao et al., 2022). Due to the decrease in secondary BA levels caused by gut microbiota dysbiosis in most patients with inflammatory diseases, it is suggested that restoring secondary BA levels by improving gut microbiota dysbiosis may help inhibit NLRP3 inflammasome activation and alleviate related diseases (Collins et al., 2023).

Additionally, BAs inhibit NLRP3 inflammasome activation by binding to receptors such as the farnesoid X receptor (FXR) and G protein-coupled receptor 5 (TGR5). FXR, a nuclear receptor, is highly expressed in tissues such as the liver, kidneys, and ileum (Adorini and Trauner, 2023). Hao et al. found that *Fxr* knockout mice were more sensitive to LPS-induced NLRP3 inflammasome-related endotoxemia, whereas *Fxr* overexpressing mice showed increased resistance (Hao et al., 2017). Further research revealed that FXR inhibits NLRP3 inflammasome activity by interacting directly with NLRP3 and caspase-1, indicating that targeted FXR activation can suppress NLRP3 inflammasome activation (Hao et al., 2017). Chen et al. found that supplementing colitis rats with *Bacteroides fragilis* containing BSH improved BA metabolism and inhibited NLRP3 inflammasome activation by activating FXR, thus reducing intestinal inflammation (Chen et al., 2024). This suggests that increasing the abundance of bacteria involved in secondary BA production can boost secondary BA levels, activate FXR, and thereby inhibit NLRP3 inflammasome activation (Figure 2).

TGR5, a transmembrane receptor, is present in various tissues including adipose tissue, skeletal muscle, intestines, and liver. Its binding with ligands leads to increased intracellular cyclic AMP (cAMP) levels, affecting downstream signaling pathways. Related experiments have shown that BAs inhibit NLRP3 inflammasome activation via the TGR5-cAMP-proteinkinase A (PKA) axis by promoting the phosphorylation and ubiquitination of the NLRP3 inflammasome (Chen et al., 2019). Additionally, activation of the TGR5-cAMP-PKA signaling pathway by DCA also inhibits NF-κB (p65) transcriptional activity, thereby reducing the expression of NLRP3 inflammasome-related proteins (Zhang et al., 2023). In mice with gut microbiota dysbiosis, impaired TGR5 activation exacerbated *Staphylococcus aureus*-induced mastitis, whereas supplementation with secondary BA-producing Clostridium restored TGR5 activation and reversed these changes (Zhang et al., 2023). Therefore, an increase in secondary BA concentration induced by bacteria that produce secondary BAs may inhibit NLRP3 inflammasome activation through the TGR5-mediated cAMP-PKA signaling pathway.

Verheggen et al. found that 8 weeks of moderate-intensity aerobic exercise increased the abundance of bacteria such as *Firmicutes* and *Ruminococcus* in the gut microbiota of obese patients (Verheggen et al., 2021). These bacteria, known for metabolizing BAs, can increase secondary BA production by enhancing the metabolism of primary BAs. Carbajo-Pescador et al. demonstrated that 5 weeks of aerobic exercise effectively reversed high-fat diet-induced Non-alcoholic fatty liver disease gut microbiota dysbiosis in rats, thereby preventing enterohepatic axis dysregulation, improving BA homeostasis, and inhibiting the expression of NF-κB and inflammatory factors (TNF-*α* and IL-6) (Carbajo-Pescador et al., 2019). Therefore, we have reason to speculate that exercise may inhibit NLRP3 inflammasome activation by increasing the abundance of bacteria associated with secondary bile acid production, elevating secondary bile acid levels, and activating FXR and TGR5-mediated signaling pathways. However, while evidence shows that exercise improves BA homeostasis and gut microbiota, their relationship is complex due to the diverse and extensive nature of gut microbiota and BA pools. This complexity makes it challenging to elucidate the precise relationship between specific BAs and microorganisms. More scientific studies are needed to advance the precise development of therapeutic targets for NLRP3 inflammasome-related diseases (Figure 2).

#### 6.2.3 Exercise regulation of trimethylamine oxide to inhibit NLRP3 inflammasome activation

Trimethylamine Oxide (TMAO) is another important metabolite derived from the gut microbiota. Choline, betaine, and L-carnitine in food are converted into trimethylamine (TMA) by the gut microbiota. TMA is absorbed by intestinal epithelial cells and transported to the liver via the portal vein, where it is oxidized to TMAO by flavin-containing monooxygenase (FMO) enzymes (Liu et al., 2016; Lang et al., 1998). Many studies have reported that elevated levels of circulating TMAO are positively correlated with the occurrence and development of cardiovascular diseases, Alzheimer’s disease, diabetes, renal diseases, metabolic dysfunction-related fatty liver, and obesity (Gatarek and Kaluzna-Czaplinska, 2021). In fact, increased levels of TMAO in the circulation can promote inflammation in various tissues, with the NLRP3 inflammasome playing a significant role in this process. Research has shown that TMAO can induce the activation of the NLRP3 inflammasome in various cell types, including vascular endothelial cells (Chen et al., 2017; Sun et al., 2016), colonic epithelial cells (Yue et al., 2017), pancreatic *β* cells (Kong et al., 2024), cardiac fibroblasts (Li et al., 2019), and renal fibroblasts (Lai et al., 2022), thereby triggering inflammatory responses. Additionally, TMAO has been shown to upregulate the expression of the macrophage surface receptor TLR4, which is a key upstream receptor for NLRP3 inflammasome activation (Hakhamaneshi et al., 2021). This suggests that TMAO may induce NLRP3 inflammasome activation in macrophages via TLR4. Therefore, targeting the suppression of metabolic pathways that synthesize TMAO may be an effective strategy for inhibiting NLRP3 inflammasome activation.

Exercise has been shown to lower TMAO levels in human circulation. For instance, Argyridou et al. tracked 483 diabetic patients over 12 months and found that moderate to vigorous exercise was linked to lower plasma TMAO levels (Argyridou et al., 2020). Similarly, Erickson et al. reported that a 12 weeks low-calorie diet combined with exercise effectively reduced plasma TMAO levels in obese adults (Erickson et al., 2019). The reduction in TMAO levels through exercise may be attributed to its positive impact on gut microbiota homeostasis. Zhang et al. demonstrated that 12 weeks of voluntary wheel running effectively reversed the effects of a TMAO diet in APP/PS1 mice, including decreased α-diversity of the gut microbiota, reduced relative abundances of *Bacteroidetes* and *Prevotella*, increased relative abundances of *Actinobacteria*, *Verrucomicrobia*, and *Ruminococcus*, and elevated serum TMAO levels. This suggests that exercise may lower TMAO levels by improving gut microbiota diversity and composition (Zhang et al., 2023b). Therefore, it is reasonable to speculate that exercise may inhibit NLRP3 inflammasome activation by regulating the homeostasis of the gut microbiota to reduce TMAO levels, which is worthy of further study (Figure 2).

## 7 Summary and prospects

In conclusion, the intricate interplay between gut microbiota and the activation of the NLRP3 inflammasome is a critical area of research with significant implications for understanding and e treating inflammatory diseases. This review emphasizes the close relationship between exercise, gut microbiota, and the NLRP3 inflammasome. It suggests that exercise can inhibit the over-activation of the NLRP3 inflammasome by regulating gut microbiota, thereby preventing and treating inflammatory diseases. Different exercise patterns have varying effects on gut microbiota and the NLRP3 inflammasome activation. Therefore, future studies are needed to further elucidate the specific mechanisms through which different exercise patterns affect gut microbiota and the NLRP3 inflammasome, as well as their potential therapeutic effects on inflammatory diseases. Additionally, individual variability in gut microbiota responses to exercise requires further investigation. Factors such as age, diet, and baseline microbiota composition likely contribute to heterogeneous outcomes, which should be considered in future research. While SCFAs, BAs, and TMAO are established metabolites involved in regulating NLRP3 inflammasome activation, other microbiota-derived metabolites (e.g., indole derivatives) require more extensive investigation. The impact of diet, which strongly influences gut microbiota, also warrants further attention. Exercise alone may not be sufficient for optimal inflammatory regulation; combined exercise and dietary interventions, such as low-carbohydrate or high-fiber diets, may offer enhanced benefits (Sun et al., 2022; Moura et al., 2024; Lin et al., 2023). Therefore, future research should focus on developing personalized exercise protocols that account for individual variability in microbiota responses, alongside integrating dietary strategies to optimize NLRP3 inflammasome regulation. These combined approaches could offer a more effective means of managing chronic inflammatory diseases, with significant implications for clinical practice and public health.
